# Rumor Surveillance and Avian Influenza H5N1

**DOI:** 10.3201/eid1103.040657

**Published:** 2005-03

**Authors:** Gina Samaan, Mahomed Patel, Babatunde Olowokure, Maria C. Roces, Hitoshi Oshitani, the World Health Organization Outbreak Response Team

**Affiliations:** * Western Pacific Regional Office of the World Health Organization, Manila, Philippines; † National Centre for Epidemiology and Population Health, Canberra, Australia

**Keywords:** avian influenza H5N1, rumor, surveillance, regional outbreak, dispatch

## Abstract

We describe the enhanced rumor surveillance during the avian influenza H5N1 outbreak in 2004. The World Health Organization’s Western Pacific Regional Office identified 40 rumors; 9 were verified to be true. Rumor surveillance informed immediate public health action and prevented unnecessary and costly responses.

In January 2004, 14 persons in Vietnam were admitted to provincial hospitals with severe respiratory illness (*1*). Avian influenza H5N1 was detected in samples from 3 of these patients. Health officials and the World Health Organization (WHO) were concerned, as these were sporadic cases of an influenza strain that normally infects birds exclusively (*2*). Furthermore, little was known about the extent of the outbreak, its potential for international spread, and the possible evolution of a pandemic influenza strain. WHO issued an international public health alert on January 13, 2004, to inform the world about the outbreak (*1*).

News of the outbreak led to international anxiety and the propagation of unofficial outbreak reports or disease rumors (*3*). These rumors could have led countries to impose trade and travel restrictions with negative social, economic, and health consequences (*3**,**4*). To protect both the international community and the affected countries, WHO introduced enhanced rumor surveillance for reports of avian influenza H5N1, a process of investigating unofficial reports of disease events to determine their veracity. Rumor surveillance aims to decrease the potential for misinformation and misunderstanding and to inform the public and health officials about disease outbreaks, facilitate a rapid response, and promote public health preparedness (*3*).

Rumor surveillance is a passive process, where rumors are identified from media reports, professional groups, the public, and persons in the WHO network, which is made up of WHO headquarters, country offices, and WHO Collaborating Centers. In an enhanced system, rumor surveillance is intensified by actively seeking out rumors and undertaking more rigorous follow up. This surveillance includes analyzing more media sources and regularly requesting information from the WHO network about outbreak events. Previous studies have examined the role of enhanced rumor surveillance during public health emergencies, such as the Chernobyl nuclear accident in 1986 and the outbreak of Ebola in Uganda in 2000 (*5**,**6*). However, research has not examined the role of rumor surveillance in multicountry or regional outbreaks.

The importance of rumor surveillance is likely to increase as the international community considers the revised draft of the International Health Regulations (IHR). Article 8 of the IHR Working Paper (*7*) states, “WHO, in consultation with the health administration of the State concerned, shall verify rumors of public health risks which may involve or result in international spread of disease.”

During the avian influenza outbreak, WHO’s Western Pacific Regional Office (WPRO) was the focal point for identifying rumors and coordinating their investigations in the region (*8*). WPRO covers 37 nations and stretches from China in the north and west, to New Zealand in the south, and to French Polynesia in the east (*9*). This study examines whether the enhanced rumor surveillance undertaken by WPRO during the first 40 days of the outbreak achieved its aims of 1) offering timely assistance to potentially affected nations, 2) prompting countries to undertake preparedness measures appropriate to their level of risk of being affected, and 3) informing the public and the international community about relevant events.

## The Study

WPRO designated a rumor surveillance officer to develop and implement the rumor surveillance system for avian influenza in animals and humans. This officer actively assessed media sources and email-based public health discussion and regularly contacted the WHO network to identify rumors. Media sources included journalists visiting WPRO and Web sites for television networks and newspapers. Most were English-based media sources; however, some were also in Japanese and Arabic. To increase the scope of the active media search, this officer also accessed the Global Public Health Intelligence Network (*10*), an electronic surveillance system that continuously monitors >600 media sources and biomedical journals in a number of languages, including Chinese, Spanish, English, and French.

Each rumor was followed up by an email or a telephone request to the relevant WHO country office to investigate its veracity. The WHO country office in turn sought verification from the country’s health authorities. Overall, the onus of the verification process was in the hands of the affected countries’ health authorities. The authorities had to demonstrate to WHO that appropriate investigations were conducted to deem rumors correct or incorrect. To ensure this process, WHO sometimes supported rumor verification by assisting in laboratory testing or shipment of isolates.

Once available, the outcome of the investigation was disseminated to WHO stakeholders, including the outbreak response team. For events reported in the media, WPRO’s media officers made information publicly available through press releases and media interviews, as well as providing up-to-date information on the WHO Web site (http://www.who.int).

From January 20 to February 26, 2004, a total of 40 rumors were identified, most within 4 weeks of the outbreak alert (Figure). The rumors concerned 12 countries and 1 special administrative region. Of the total rumors received, 19 (48%) were received from the media, 18 (45%) from the WHO network, 2 (5%) from embassy staff living in affected countries, and 1 (2%) from ProMED Digest with a media source as the origin. Nine (23%) rumors were confirmed to be true events: 5 in China and 1 each in Cambodia, Japan, Laos, and South Korea. Of the incorrect rumors, 6 were in China, 6 in Laos, 4 in Vietnam, 4 and in Hong Kong, 3 in Cambodia, 2 in Germany, and 1 each in Bangladesh, Indonesia, Japan, Malaysia, Saudi Arabia, and Singapore.

**Figure:**
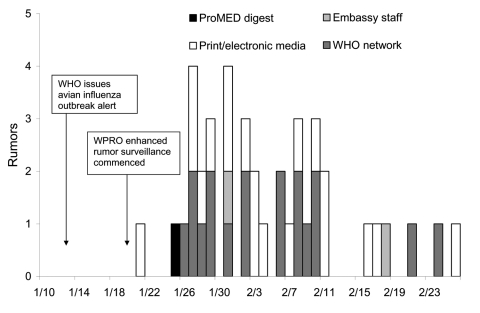
Number of rumors received from January 20 to February 26 by source of rumor, Western Pacific Regional Office (WPRO) of the World Health Organization (WHO), 2004.

The average period for verification of true events was 2.7 days (range 1–5 days). The average period to verify that a rumor was incorrect was 9.3 days (range 1–26 days). Sixty percent of the rumors related to human outbreaks, of which 1 was true, and 40% to animal outbreaks, of which 8 were true. The Table provides examples of rumors received during the 40-day study, the outcomes of the investigation, and the public health action taken. The remaining 32 rumors are not shown for reasons of brevity and privacy; however, not all rumors resulted in public health action after the verification process. This finding was expected because the high sensitivity of the system decreased the predictive value positive.

**Table:** Avian influenza H5N1 rumors, Western Pacific Regional Office of World Health Organization (WHO), 2004

Rumor (source, date)	Verification outcome (verification date)	Public health action
A poultry farm outside Phnom Penh had 500 chicken deaths with no identified cause (Dow Jones International News, 1/21/04).	True. Poultry deaths result of avian influenza H5N1 infection (1/24/04).	Thailand banned importation of poultry from Cambodia (1/24/04). Japan supplied stocks of oseltamivir to be used as prophylaxis. WHO supplied personal protective equipment for people involved in culling poultry within a 5-km radius of the affected poultry farm.
Duck deaths in Guangxi, China, with no identified cause (WHO network, 1/26/04).	True. Avian influenza H5N1 confirmed (1/28/04).	48 countries banned importation of poultry from China (South China Morning Post, 1/29/04). WHO invited 2 Dutch experts to assist China contain the outbreak.
14-year-old boy died from respiratory illness in Hong Kong after returning from Guangdong, China (Wenhui Newspaper, 8/2/04).	Incorrect. Negative influenza A and SARS tests (21/2/04)	Hong Kong reported outcome of the investigation in the media but no public health action was taken (21/2/04).
Persons in 2 Laotian provinces who ate chicken died of natural causes (WHO Network, 2/6/04).	True (2/11/04).	WHO fast-tracked and released draft guidelines on food safety (2/12/04). These guidelines were distributed to ministries of health and to other health authorities, and posted on the WHO Web site (http://www.who.int/foodsafety/micro/avian2/en/).
Four pigs tested positive for avian influenza H5N1 in Vietnam (Reuters Health Online, 2/6/04).	Incorrect. Virus isolated from nasal swabs not indicative of influenza infection (2/6/04).	Media officers from WHO and the Food and Agricultural Organization independently held media releases to emphasize outcomes of the investigation (2/7/04).
An outbreak of avian influenza H5N1 in a poultry farm 100 km south of Seoul, Korea (South China Morning Post, 1/27/04).	True (1/28/04).	Virus from the outbreak was analyzed by WHO network laboratories to determine the susceptibility of the strain to antivirals. The strains tested demonstrated in vitro susceptibility to oseltamivir (http://www.who.int/csr/don/2004_02_12a/en/).
Human case of avian influenza in a German tourist returning from Asia (Washington Times, 1/22/04).	Incorrect (1/24/04).	WHO issued a press release on the outcome that infection had not spread internationally, so there was no need to shift into the Influenza Pandemic Plan Phase 1 (1/24/04 and 1/26/04).
48 children sick with respiratory illness in Nam Dinh Province, Vietnam (WHO network, 8/2/04).	Incorrect. Negative influenza tests (9/2/04).	No action taken after the rumor verification process was completed.

## Conclusions

WPRO’s enhanced rumor surveillance system identified many rumors. Most were identified in the first few weeks after the public health alert. A similar pattern was also observed during the 2003 SARS outbreak, when most rumors were received within the first 7 weeks of the public health alert (*11*). The decreased rate of rumor detection later in the outbreak is consistent with Allport and Postman’s basic law of rumor (*12*). According to this law, the amount of rumors in circulation is roughly equal to the importance of the rumor multiplied by the uncertainty surrounding the rumor. We found that, as more information became available about the outbreaks and about the H5N1 virus, fewer rumors circulated. This decrease was despite the fact that the importance of the disease remained high because of the ongoing risk for evolution of a pandemic influenza strain.

Through rumor surveillance, WHO assisted affected countries by issuing guidelines, providing technical expertise, and mobilizing supplies. Unaffected countries also took action by banning the importation of poultry from affected countries. This action was crucial in preventing the further spread of avian influenza.

An important part of rumor surveillance is the timely dissemination of accurate information to reduce misunderstanding and unwarranted concern, especially for rumors reported in the media. One example was the need to address the international concern that arose about the rumor that pigs were infected with avian influenza (*13*). If the rumor had not been reported to be incorrect publicly after the verification process, health authorities may have heightened avian influenza surveillance to include the investigation of persons with symptoms of influenza and a history of contact with pigs.

The literature lacks guidance on how to establish and operate enhanced rumor surveillance during large outbreaks. Based on our experience and drawing on the recommendations in standard texts on public health surveillance (*14**,**15*), we suggest the following criteria for developing rumor surveillance: 1) Define the goals of surveillance as part of an early warning system in which each rumor deserves investigation to determine its veracity; 2) Apply a case definition that will have a high level of sensitivity (and therefore a relatively lower specificity) to identify the event of interest early in the outbreak; 3) Articulate clearly the steps to be undertaken to assess the veracity of the rumor, the criteria for deeming the verification process complete, and the ethics and confidentiality in conducting investigations; 4) Clarify the actions to be taken if the rumored events are true, or incorrect, or if the response of the verifying authority lacks credibility; 5) Delegate responsibility for data collection, management of the rumor database, and verification to a person trained in surveillance. This person must have access to relevant national and international networks and appropriate negotiation skills to investigate the veracity of the rumors. In selected instances, multilingual staff may be essential; 6) Include among the data sources print and electronic media, the Global Public Health Intelligence Network, national health authorities, and professional bodies and networks. Consider mechanisms for the public to report rumors through a hotline or an email address; 7) Develop mechanisms to provide regular updates on current verification activities, the number of rumors investigated, and their outcomes to the outbreak response team; 8) Provide regular feedback on the outcomes of investigations to those who provided data, and where appropriate, to the international community; and 9) Evaluate the efficiency and effectiveness of the investigations and upgrade the rumor surveillance system through a process of continuous quality improvement.
